# High Intake of Manganese During Second Trimester, Increases the Risk of Preterm Delivery: A Large Scale Cohort Study

**DOI:** 10.5539/gjhs.v7n5p226

**Published:** 2015-03-16

**Authors:** Sare Bakouei, Fatemeh Reisian, Minoor Lamyian, Ebrahim Haji Zadeh, Hadi Zamanian, Zahra Taheri Kharameh

**Affiliations:** 1 Faculty of Nursing and Midwifery, Qom University of Medical Sciences, Qom, Iran; 2 Reproductive Health & Counseling in Midwifery Research Centre, Golestan University of Medical Sciences, Gorgan, Iran; 3 Department of Midwifery and Reproductive health, Tarbiat Modares University, Tehran, Iran; 4 Department of Medical Science, Tarbiat Modares University, Tehran, Iran; 5 Department of Health Education and Promotion, School of Public Health, Tehran University of Medical Sciences, Tehran, Iran; 6 School of Paramedical Sciences, Qom University of Medical Sciences, Qom, Iran

## Abstract

Evidence indicates that nutrients and minerals might play an important role in preterm delivery (PTD). The aim of this study was to determine maternal nutritional status during second trimester of pregnancy and its association with preterm delivery (< 34 weeks gestation) in Iranian women. In a large scale longitudinal study, 1033 pregnant women were recruited from prenatal clinics since December 2012 to June 2013. Dietary intake was assessed by Food Frequency Questionnaire (FFQ) in pregnant women of 14 to 20 weeks gestational age. The participants were followed up until delivery. Dietary intake of women with preterm delivery was compared with women who had term delivery. The results show that 61.2% of women were primiparous and that the incidence of preterm delivery was 7%. Manganese dietary intake was significantly higher in mothers with preterm delivery than those with term delivery (P = .03). Manganese was the only micronutrient correlated with preterm delivery after adjustment for maternal characteristics during second trimesters of pregnancy (OR = 1.12; P = .01). These results suggest that high maternal manganese dietary intake during the second trimester of pregnancy may be associated with the risk of preterm delivery in Iranian pregnant women.

## 1. Introduction

The importance of proper nutrition before and during pregnancy has long been known for maternal, fetal and child health (Mistry & Williams, 2011). Pregnancy is a natural phenomenon with increased metabolic demands and changes in maternal physiology and the dietary supply (Al-Shoshan, 2007). Dietary intake of nutrients and maternal nutrition affects the availability and supply of nutrients to fetus.

Inadequate intake of nutrients can have adverse effects on pregnancy outcomes such as increasing maternal morbidity and mortality (Keen et al., 2003; Tabrizi & Pakdel, 2014). Nutritional deficiencies, especially micronutrient deficiencies during early pregnancy has been related to low birth weight, preterm delivery and other perinatal outcomes (Ramakrishnan et al., 2012).

Preterm birth (PTB) is a global health problem and the leading risk factor of perinatal morbidity and mortality (Zhang et al., 2012). It is defined as an infant born at less than 37 weeks gestational age. In Iran, the incidence of preterm delivery has been reported 15.5% (Dolatian et al., 2014). Preterm birth is responsible for 70% of neonatal deaths and 75% of neonatal morbidity. It is a major cause of short and long term neonatal damages including neurocognitive deficits, ophthalmological disorders and pulmonary dysfunction in surviving preterm infants (Wen et al., 2004).

Several studies investigated the association between Various nutrients or elements and preterm birth. Mirzarahimi et al reported that maternal zinc concentration has no impact on neonatal birth weight (Mirzarahimi et al., 2011). Ronnenberg et al. found that elevated level of homocysteine during the preconceptional period was associated with the higher risk of PTD (Ronnenberg et al., 2002). In another study, Bukowski et al. reported that preconceptional folate supplementation that was prospectively recorded in the first trimester of pregnancy was associated with significant reduction in the incidence of PTD (Bukowski et al., 2009). In the Danish National Birth Cohort study, Catov reported that Regular periconceptional multivitamin use was associated with reduced risk of PTD in non overweight women (Catov et al., 2011). However, to the best of our knowledge, there is no longitudinal study regarding the association between the maternal nutritional status and preterm delivery in Iranian women. For this reason, the aim of our study was to examine the maternal dietary intake in pregnant women during second trimester and its association with preterm delivery in Iran in a large sample cohort study.

## 2. Materials and Methods

### 2.1 Design and Participants

This longitudinal study was conducted between December 2012 to June 2013 on pregnant women recruited at prenatal health centers in Tehran, Iran. The pregnant women referred to main prenatal centers were selected using a multistage sampling method according to the following inclusion criteria: being aged 16 years or older, living in Iran, carrying a singleton pregnancy with gestational age (GA) between 14 to 20 weeks, normal course of pregnancy and ability to communicate in Persian. Exclusion criteria was chronic diseases such as cardiovascular disease (CVD), diabetes mellitus, renal disease, and hypertension.

Women who met the inclusion criteria and signed the informed consent were eligible to participate. After enrollment, by a face to face interview of well-trained raters, information about pregnancy history, demographic and background data and dietary intake by food frequency questionnaire (FFQ) were collected. Participants were followed up until labor. Mothers who delivered at less than 37 weeks were assigned to the preterm group and the remained with delivery at GA equal to or greater than 37 weeks assigned to the term group.

### 2.2 Instruments

Dietary intake was assessed by food frequency questionnaire (FFQ). The FFQ is considered as an acceptable tool to assess dietary intake in large surveys, including prenatal studies. The FFQ assess the usual frequency of foods consumption (60 item) over the month prior to the interview date. Frequency of consumption of each item was categorized as never, once per day, 2 to 3 times per day, 4 or more times per day, once per week, 2 to 4 times per week, 5 to 6 times per week. For analysis purposes, all food frequencies were transformed into times per day. The daily frequencies were multiplied with standard portion sizes to calculate food intake in grams (Malekshah et al., 2006). The calculation of nutrient intake was performed in nutritionist version 4 software.

In addition, information about socio-demographic and pregnancy history such as age, maternal education, employment and economic status, gestational age, gravidity, parity, abortions and any complications in previous pregnancies, use of vitamin or mineral supplements was obtained.

### 2.3 Data Analysis

Descriptive results were shown as means ± SD or as numbers and percentages. Differences in dietary intake between PTD group and term group were analyzed by Independent t-test. Logistic regression was used to assess the relationship between maternal dietary intake and the PTD after adjusting for the potential confounders. Statistical analyses were performed using SPSS version 16. Statistical significance was assumed at p<0.05.

### 2.4 Ethical Considerations

Ethical approval was given by the Ethics Committee, Tarbiat Modares University. The participants were provided with information about the study process. Signed consent forms was obtained from all subjects and they agreed to be the subjects until delivery. Participation in this study was free to withdraw from the study at any time.

## 3. Results

A total of 1033 women participated in the study and completed the follow up with a mean age of 26.7 ± 4.3 years. 61.2% of women were primiparous. Further demographic and pregnancy information is listed on Table 1.

**Table 1:** Socio-demographic and pregnancy information of the participants (N = 1033)

Categorical variables	Participants	

	Number, N	Percentage, %
**Age group, years**		
18-25	423	40.9
26-35	610	59.1
**Education**		
Illiterate	12	1.2
High school	741	71.7
University	280	27.1
**Job**		
Housewife	896	86.7
Employer	94	9.1
Worker	1	0.1
Other	42	4.1
**Gravidity**		
<2	551	53.3
2-4	436	42.2
>4	46	4.5
**Parity**		
0	632	61.2
1-3	400	38.7
3<	1	0.1
**Abortion**		
0	852	82.5
1-3	179	17.3
>3	2	0.2
**Supplement**		
**Iron**		
Yes	588	56.9
No	445	43.1
**Multivitamin**	
Yes	355	34.4
No	678	65.6
**Calcium**		
Yes	155	15
No	877	84.9
**Folic acid**		
Yes	958	92.7
No	75	7.3
**History of PTD**		
Yes	27	2.6
No	1006	97.4

After follow up period (delivery), 72 of 1033 pregnancies (7 percent) led to preterm delivery (PTD) and the remained assigned to term group. Table two shows the mean intake of micronutrients and macronutrients in the pregnant women in total subjects and both PTD and term groups. Except for iron, folate, vitamin E, vitamin D and potassium intake, the dietary intake of all macronutrients and micronutrients was higher than the Recommended Dietary Allowance (RDA). The lowest intake was of iron, vitamin E and vitamin D (below 50% of RDA).

The results of Independent t-test showed a statistically significant difference in manganese dietary intake between preterm and term groups (P = .03). Manganese dietary intake was significantly higher in mothers with PTD than term delivery mothers (Table 2).

**Table 2:** Daily energy, macronutrient, and micronutrient intake of mothers in preterm and term groups

	Preterm group n=72 Mean ± SD	Term group n=961 Mean ± SD	Total n=1033 Mean ± SD	RDA	P-value*
Energy, kcal	2070±956	1980±995	1933±790	2050	0.51
Protein, g	83.33±38.45	83.52±46.73	83.50±46.18	66	0.93
Carbohydrate, g	334±176	329±174	329±184	175	0.65
Fat, g	49.78±25.77	49.78±26.22	49.78±26.18	35	0.78
Cholesterol, mg	259±176	256±184	256±159	250	0.88
Ca, mg	1081±626	998±535	1004±544	800	0.18
P, mg	964±372	952±354	958±318	700	0.21
Mn, mg	3.60±3.21	2.99±2.35	3.03±2.42	2	0.03*
iron, mg	16.48±11.36	17.01±14.54	16.97±14.34	34	0. 76
Zn, mg	9.12±5.28	8.98±4.17	8.99±4.25	6.6	0. 78
K, mg	3488±1825	3382±1704	3401±1782	4700	0. 61
Vit B-1, mg	1.10±0.51	1.24±0.72	1.15±0.65	1.4	0. 91
Vit B-2, mg	1.28±0.71	1.18±0.61	1.21±0.81	1.7	0. 45
Vit B-3, mg	13.4±5.8	12.6±6.2	13.1±6.8	18	0. 86
Vit B-6, mg	4.57±6.17	5.10±6.23	4.97±7.17	1.9	0. 37
Vit B-12, μg	6.18±5.88	6.24±8.36	6.23±8.21	2.6	0. 95
Folate, μg	329±227	294±190	296±193	800	0.13
Vit C, mg	152±117	150±122	150±121	80	0. 91
Vit A, μg	1406±1356	1216±1181	1230±1194	800	0.19
Vit E, mg	3.72±3.69	3.11±2.59	3.16±2.68	12	0.06
Vit D, μg	2.15±2.12	1.9±2.10	1.95±2.10	5	0.41

* p-value less than 0.05.

We applied multiple logistic regression to estimate the association between dietary intakes and the risk of preterm birth while controlling the effect of potentially confounding variables. Dietary intake as well as the socio-demographic and pregnancy variables were entered in multiple logistic regression model. A significant relationship were seen between manganese dietary intake and preterm birth after controlling the other variables [OR = 1.12 CI: (1.02-1.23), p = 0.01)] (Table 3).

**Table 3:** Logistic regression analysis of Preterm and maternal Dietary intake

Maternal dietary intake	Preterm		

	OR	*P*	Interval confidence %95 CI
Energy, kcal	1	.29	.99-1
Protein, g	.99	.47	.99-1.01
Carbohydrate, g	.98	.62	.99-1
Fat, g	.99	.48	.97-1.02
Cholesterol, mg	1	.98	.99-1
Ca, mg	1.01	.08	1-1.02
P, mg	.91	.41	.95-1
**Mn, mg**	**1.12**	**.01**	**1.02-1.23**
Fe, mg	.95	.85	.56-1.61
Zn, mg	.96	.47	.86-1.07
K, mg	1	.42	.99-1
Vit B-1, mg	1.19	.51	.70-2.02
Vit B-2, mg	1.12	.39	.86-1.45
Niacin, mg	.98	.81	.97-1.01
Vit B-6, mg	1.02	.28	.97-1.07
Vit B-12, μg	.98	.58	.94-1.05
Folate, μg	1	.27	.99-1
Vit C, mg	.99	.29	.99-1
Vit A, μg	1	.56	.99-1
Vit E, mg	1.08	.05	.99-1.12
Vit D, μg	.87	.98	.85-1.14

Adjusted for maternal age, gestational age, education, parity, gravidity, maternal BMI, income, pregnancy history and use of supplements.

## 4. Discussion

The results of our longitudinal study indicated that high manganese dietary intake of pregnant women is associated with preterm birth. Manganese is one of the essential nutrients for human and animal. It is required for development and function of skeletal systems, reproductive hormones, enzymes activation and cell protection by antioxidant function. However, some studies indicate toxicity effects of high manganese on fetus during pregnancy (Wood, 2009).

The effects of manganese intake on fetal development and pregnancy outcome have been an area of interest in animal studies, but little information is available with respect to the specific roles of manganese in birth outcomes in humans (Vigeh et al., 2008; Zota et al., 2009; Eum et al., 2014). Vigeh et al. (2008) report that blood manganese is associated with the risk of intrauterine growth retardation (IUGR). In a cohort study, Eum et al. (2014) found that both extremes of maternal manganese levels is associated with lower birth weight outcome in Korea. Zota et al. (2009) found a relationship between blood manganese and birth weight in a cohort of 470 mother-infant pairs from Oklahoma.

In the present study, the Iron and Folate dietary intake from food was lower than the recommended energy and nutrient intakes. Public health interventions about increasing iron intake during early pregnancy whether from diet or supplements, need to be promoted. Strengths of this study include the large sample size of this population cohort study (n = 1033) and the collection of detailed information on diet in second trimester of pregnancy which allowed a comprehensive assessment of micronutrients and macronutrients intakes. As well, our report on effect of high manganese intake on PTD is almost new and could be followed by some other studies on manganese serum level and related mechanism of effect. The limitation of this study is that dietary intake is not evaluated in first and third trimesters and delivery time which reduce generalizability of the findings of this study. Future studies are suggested to remove this limitation and consider serum manganese along with its intake with relation to PTD and other birth outcomes.

## 5. Conclusion

Most participants report insufficient iron, folate, vitamin E and vitamin D dietary intake from food. Prenatal clinicians should encourage these supplementation and diet among pregnant women during early pregnancy. High maternal manganese dietary intake was associated with increased risk of PTD. Manganese is one of the least studied micronutrients, so much attention is needed in nutritional consultations about possible risk of excessive intake of manganese, however, future research related to the role of manganese exposure in PTD and other birth outcomes seems to be necessary.
